# Functionalized iron oxide nanoparticles: synthesis through ultrasonic-assisted co-precipitation and performance as hyperthermic agents for biomedical applications

**DOI:** 10.1016/j.heliyon.2022.e09654

**Published:** 2022-06-06

**Authors:** L.M. AL-Harbi, Mohamed S.A. Darwish

**Affiliations:** a Chemistry Department, Faculty of Science, King Abdulaziz University, P.O. Box 80203, Jeddah, 21589, Saudi Arabia; b Egyptian Petroleum Research Institute, 1 Ahmed El-Zomor Street, El Zohour Region, Nasr City, Cairo, 11727, Egypt

**Keywords:** Functionalized iron oxide nanoparticles, Specific absorption rate, Hyperthermia, Biomedical applications

## Abstract

Dual-functional iron oxide nanoparticles (IONPs), displaying self-heating and antibacterial effects are highly desired for biomedical application. This study involved the synthesis of functionalized IONPs coated with 3-aminopropyltriethoxysilane and polyethylene glycol via ultrasonic-assisted co-precipitation technique. The synthesized IONPs were then characterized by using Fourier-transform infrared spectroscopy, X-ray diffraction, dynamic light scattering, scanning electron microscopy, zeta potential, vibrating sample magnetometer and thermogravimetric analysis techniques. In addition, the effect of the synthesized IONPs on bacterial growth (*S. aureus* and *E. coli*) was studied. The influence of magnetic field power, as well as the viscous carriers on the heating efficiency of the synthesized IONPs was investigated. The specific absorption rate values increased as the power increased and decreased with the increase in the carrier viscosity. These characteristics render the synthesized iron oxide nanoparticles synthesized in the present study suitable for biomedical application as hyperthermic agents.

## 1 Introduction

Iron oxide nanoparticles (IONPs) are widely applied in biomedical applications as they attain both high biocompatibility and superparamagnetic performance [1, 2, 3, 4, 5]. IONPs could be regarded as good heat mediators and drug delivery agents in the hyperthermia-based treatment of cancer [1]. IONPs may also serve in magnetic resonance imaging as diagnostic and therapeutic agents. IONPs exhibit magnetic induction heating, which makes them suitable for several biomedical applications, including their application in localized drug delivery and purification systems as magnetically separable materials [6, 7]. The magnetic induction heating performance of IONPs relies mainly on their properties and the conditions of an externally applied magnetic field. Owing to the interaction between gradient magnetic field and magnetic moments produced by the nanoparticles, the magnetic behavior develops. Through utilizing IONPs, the energy of alternating magnetic field (AMF) is converted into heat via relaxation processes which are Brownian relaxation as well as Néel relaxation [6]. Néel relaxation occurs because of the reorientation of magnetization, arising from the reorientation that takes place within the core against the energy barrier. On the other hand, Brownian relaxation occurs due to the rotational diffusion of the particle within the carrier. Hyperthermia may occur on the IONPs, and it is possible to influence this phenomenon by using an AMF as the release of heat relies on the oscillation of the IONPs based on the processes of Brownian and Néel relaxation [6]. Hyperthermia in living tissues takes place as the temperature of tissue elevates above the values that are considered physiologically normal. Hyperthermia is applied for killing cancer cells in cancer therapy. It is also applied in the induction of local drug release from thermo-sensitive vehicles [2, 3]. Regarding the artificially-induced hyperthermia, it includes the local elevating of the temperature of the target cell up to 42 °C, facilitating the specific targeting and killing of the cancer cells, without affecting the surrounding healthy cells [8, 9, 10, 11, 12]. Unmodified IONPs, having a large surface/volume ratio and, as a consequence exhibit particle aggregation. The colloidal stability and aggregation of nanoparticles lead to a loss of the size-dependent behavior of nanoparticles like superparamagnetic character. The outstanding potential of functionalized iron oxide nanoparticles arises from their ability to gain structure with the integration of properties that neither individual material has. Functionalized IONPs are designed based on the principle of size and surface area which is associated with much higher reactivity. The introduction of functional layers on the IONPs surface shields the core of magnetic iron oxide from oxidation in chemical environment or physiological fluid. Moreover, these layers result in enhanced colloidal stability and compatibility of the nanoparticles in the physiological environment. Since the coating may affect the biocompatibility of the IONPs, the careful selection of both appropriate coating material and the coating procedure is critical to prevent any side effects on the biocompatibility of IONPs. The surface functionalization of the IONPs may be achieved by fabricating an organic coating through a physical or chemical reaction which would generate IONPs for advanced applications. Surfactants and polymers could serve as stabilizer, via steric effects and electrostatic repulsions, for enhancing the stability. Upon utilizing this approach, many polymer-shelled and organic material-shelled IONPs have been fabricated [6, 7]. Various techniques are available for IONP fabrication, such as thermal decomposition, hydro-solvothermal and co-precipitation techniques [9, 10]. Among these, the co-precipitation technique is the most widely studied one due to its simplicity, environment-friendliness as well as amenable operation at approximately low temperatures. IONPs which were synthesized using the co-precipitation process have demonstrated antibacterial and self-heating properties. In a study, the magnetic heating specific absorption rates and the antibacterial performance of three sets of these nanoparticles were evaluated. It was concluded that the concentration of 150 mg/L of these nanoparticles resulted in 10% the growth inhibition of *S. aureus* and *E. coli* [11]. Regarding another study, poly acrylic acid-IONPs -g- Kappa carrageenan nanocomposite was found to serve as a promising *in-vitro* antibacterial agent with drug efficiency 105 ± 8 μg/mg [12]. APTES and PEG are hydrophilic materials with good biocompatibility and low toxicity, properties which render these suitable for medical applications. In this study, IONPs were prepared with dual-action self-heating and antibacterial properties using the ultrasonic-assisted co-precipitation technique. The properties of the bare IONPs, APTES@IONPs, and PEG@IONPs, including the particle size, surface charge, morphology and particle phase were investigated. The effects of the synthesized IONPs on bacterial growth rates were studied. Additionally, the impacts of magnetic power, carrier viscosity and induction time on the heating efficiency and specific absorption rate (SAR) values of the synthesized IONPs were determined.

## 2 Experimental procedure

### 2.1 Materials

Iron (II) chloride tetrahydrate (purity ≥99%), Iron (III) chloride hexahydrate (purity ≥98%), ammonium hydroxide (26%), polyethylene glycol (PEG6000; purity ≥95%), and 3-aminopropyltriethoxysilane (APTES; purity ≥97%) were obtained from Sigma-Aldrich.

### 2.2 Synthesis of bare iron oxide nanoparticles (IONPs)

1.9 g of FeCl_2_.4H_2_O and 5.4 g of FeCl_3_.6H_2_O were mixed by using in 2:1 as a molar ratio of Fe^3+^/Fe^2+^. The mixture was then dispersed in 100 mL of deionized water and heating the solution to 70 °C. Immediately, ammonium hydroxide (6 mL) was added to the mixture, followed by stirring well of the resulting suspension at 70 °C for 30 min. The distilled water was used many times to wash the product, leaving behind the iron oxide nanoparticles, which were then dried at 40 °C (25 mbar) in a rotary evaporator until the powder was obtained.

### 2.3 Synthesis of the APTES-modified iron oxide nanoparticles (APTES@IONPs)

The iron oxide solution (25 mL, 0.0128 M), prepared in the previous experiment, was diluted to a volume of 150 mL using absolute ethanol and deionized water (1 mL). The diluted solution was treated for 1 h in sonicated bath (28 kHz at 25 °C). APTES (35 μL) was added with rapid stirring for 2 h. The ethanol was used to wash the product, leaving behind the APTES@IONPs, which were dried at 40 °C (25 mbar) in a rotary evaporator until the powder was obtained.

### 2.4 Synthesis of the PEG-modified iron oxide nanoparticles (PEG@IONPs)

PEG@IONPs were prepared by dissolving FeCl_2_.4H_2_O (1.99 g) and FeCl_3_.6H_2_O (3.24 g) in deionized water (50 mL) (Beaker 1), and ammonium hydroxide (30 mL) in 50 mL of deionized water (Beaker 2). PEG (2.5 g) was dispersed in deionized water (100 mL), and the resulting solution was mixed well. 25 mL of PEG solution was added to beaker 1 and beaker 2, followed by stirring in sonicated bath (28 kHz at 25 °C) to get a homogenous solution. The deionized water was utilized to wash the product, leaving behind the PEG@IONPs, which were then dried at 40 °C (25 mbar) in the rotary evaporator until the powder was obtained.

### 2.5 Bacterial growth rate

The bacterial inoculum was fabricated from a single colony cultured at 37 °C overnight in a soya nutrient broth. The culture was performed to an optical density at 600 nm value of 0.01–0.02 by utilizing the DR6000 UV–Vis spectrophotometer.

### 2.6 Statistical analysis

Through performing ANOVA in GraphPad Prism software, all the results were analyzed. Dunnett's multi-comparison test was utilized for comparing the differences in the mean growth rate values between *E. coli* and *S. aureus*.

### 2.7 Characterization

Tensor 27 Infrared Spectrometer (Bruker, USA) was utilized to perform Fourier-Transform Infrared Spectroscopy (FT-IR). Zetasizer Nano analyzer (Malvern Instruments, USA) was utilized for performing the zeta potential measurements. Dynamic Light Scattering (DLS) analysis was obtained in a Zetasizer Nano DLS unit. X-ray diffraction (XRD) was performed using a Pan Analytical Model X'Pert Prob equipped with CuKα radiation (λ = 0.1542 nm). Scanning electron microscopy (SEM) was performed by using a Zeiss ULTRA Plus field-emission SEM equipped with a Schottky cathode. (Zeiss, Germany). Thermogravimetric Analysis (TGA) was performed with the TA Instrument Q500. The magnetic behaviors were determined by vibrating sample magnetometer (VSM; Lake Shore 7400 series; Lake Shore Cryotronics, USA) with calculated solid samples at room temperature. The heating profile of the specimens were evaluated using Cheltenham induction heating limited (CIHL) with a frequency of 142 kHz, the power of 1 and 0.5 kW and a field strength of 30 kA/m. The infrared thermometer served for measuring the temperature. The SAR measurements were obtained from the first 10 s at a particle concentration of 0.2 g/mL (Eq. (1)).

(1) *SAR = (C*_*p*_*/m) × (dT/dt)*

*dT/dt*: temperature per time, *Cp*: 4.184 and *m*: the weight.

## 3 Results and discussion

### 3.1 Properties of nanoparticles

**Figure 1:**
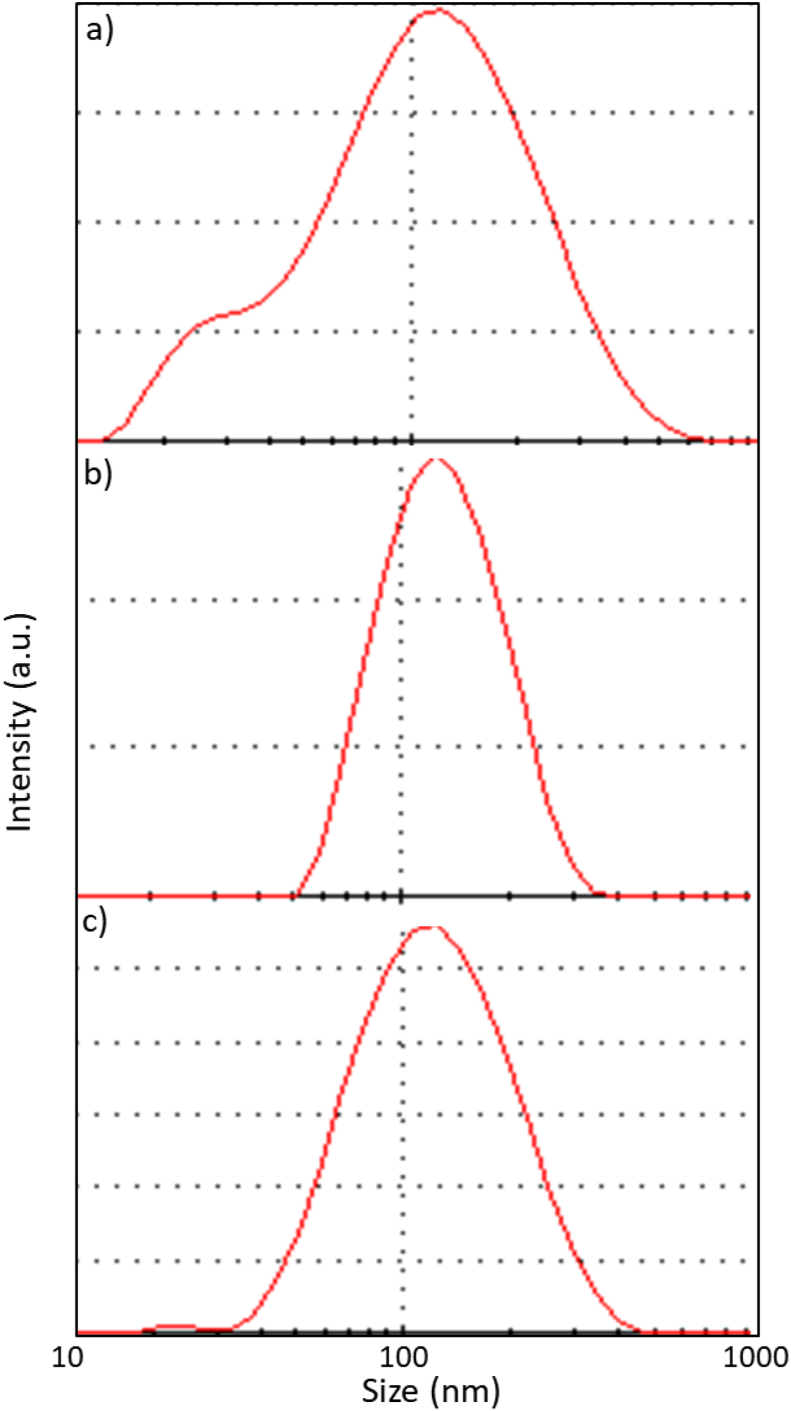
DLS results for a) IONPs, b) APTES@IONPs and c) PEG@IONPs.

The size distribution and the mean size of the synthesized nanoparticles were determined through DLS [13]. The results of the DLS analysis for the bare IONPs, APTES@IONPs and PEG@IONPs are presented in Figure 1. The mean hydrodynamic sizes of the bare IONPs, APTES@IONPs, and PEG@IONPs were 89, 123, and 109 nm, respectively. The synthesized bare IONPs exhibited a wider size distribution compared to the APTES@IONPs and PEG@IONPs. The presence of PEG polymeric layer or an organic APTES shell on the IONPs surface increased the size of the synthesized coated IONPs with a narrow distribution range.

**Figure 2:**
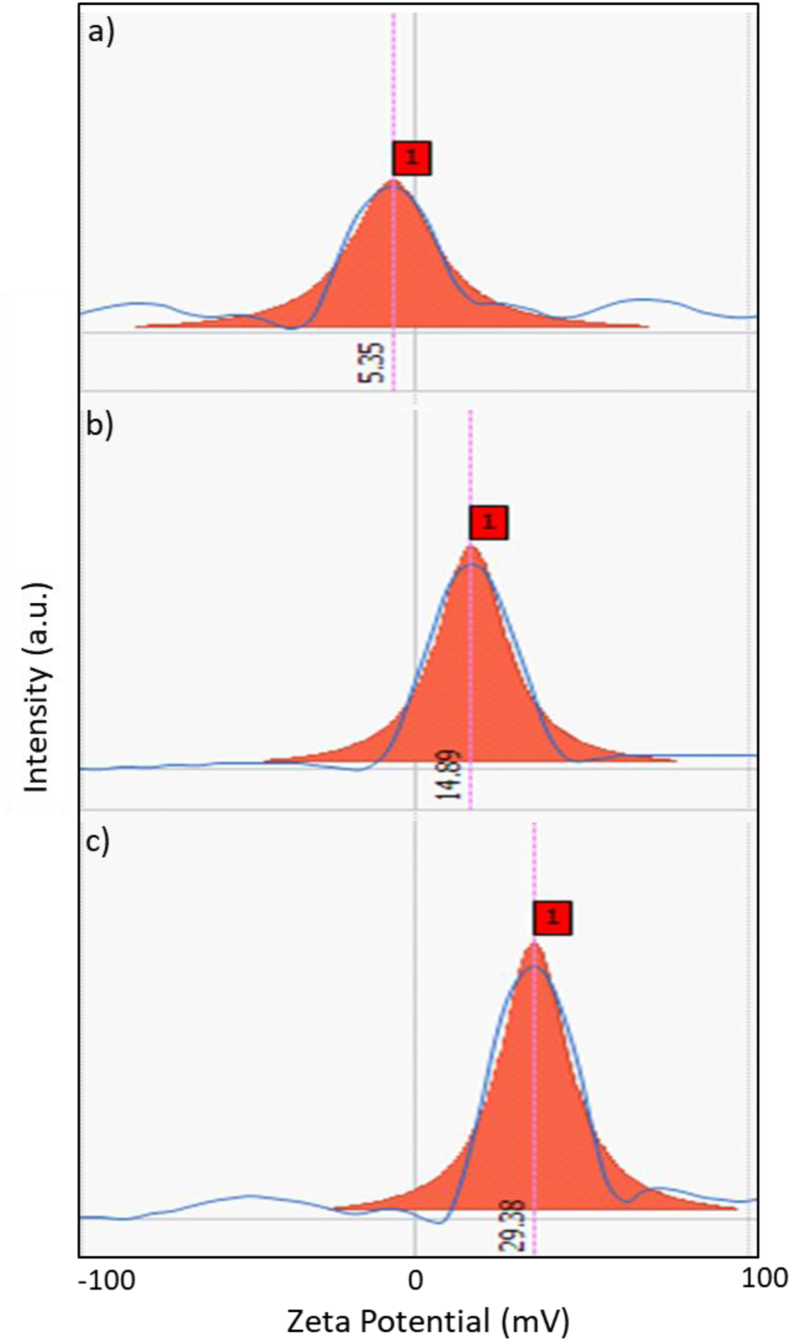
Zeta potential values for a) IONPs, b) APTES@IONPs and c) PEG@IONPs.

The zeta potential (ζ), the measure of colloidal stability and aggregation of nanoparticles, was evaluated for the synthesized nanoparticles in this study (Figure 2). The stability of nanoparticles is a crucial factor for achieving the desired and consistent outcomes in their biomedical application [14]. In this work, the synthesized uncoated IONPs exhibited a low zeta potential value of +5.35 mV. This low value indicated that these IONPs might exhibit poor stability in aqueous solutions. Low zeta potential values (0 to ±5 mV) reflect improved van der Waals inter-particle attractions, which then lead to rapid coagulation or flocculation of the nanoparticles. On the contrary, the coated iron oxide nanoparticles APTES@IONPs and PEG@IONPs exhibited high zeta potential values of +14.89 and +29.38 mV, respectively. These higher values indicated that the coated iron oxide nanoparticles would exhibit good stability in aqueous solutions. Stabilizers with a PEG polymer layer or an organic APTES layer could serve as a shell around the surface of the nanoparticle for obtaining higher stability via the steric effects and electrostatic repulsions. The value of ≈±30 mV for zeta potential is reported to reflect the stability of nanoparticles which could owe to the present high electrostatic repulsive forces among the nanoparticles [14].

**Figure 3:**
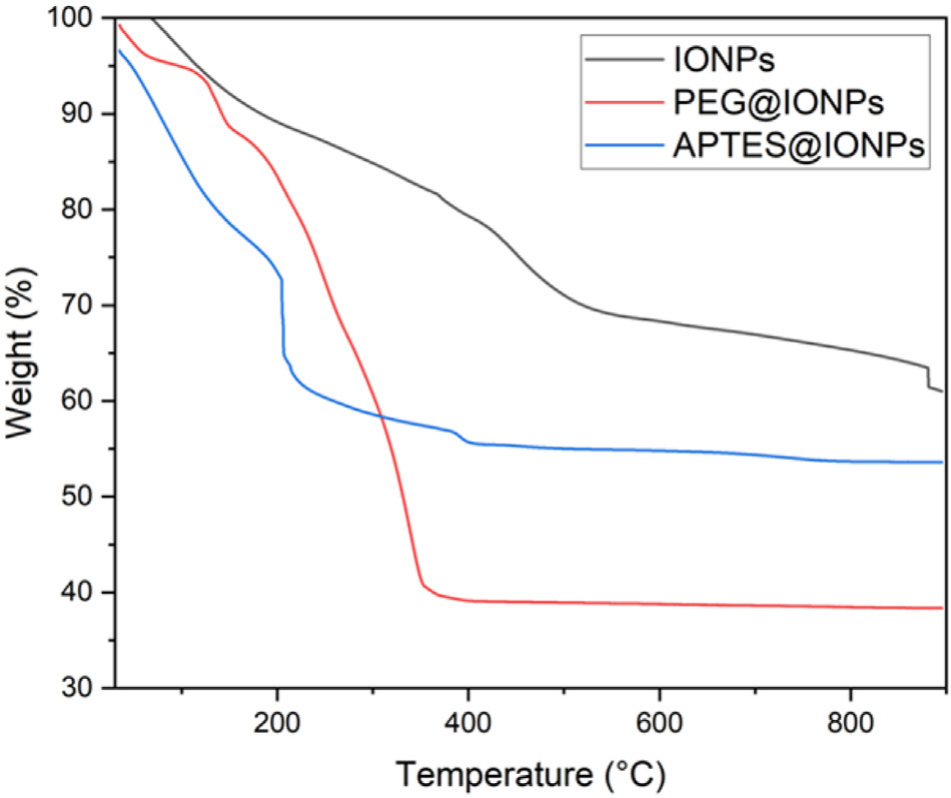
TGA results for IONPs, APTES@IONPs and PEG@IONPs.

The thermal stability of the uncoated IONPs, APTES@IONPs and PEG@IONPs was evaluated through using thermogravimetric analysis (TGA). The results are presented in Figure 3. The weight loss that occurs around 200 °C is associated with the water and impurity phase content as unwashed salts. Above 200 °C, the APTES and PEG shell materials decompose. According to the results, APTES@IONPs and PEG@IONPs exhibited the onset of decomposition at a temperature lower than that observed for the uncoated IONPs, which arises from the presence of the organic and polymer layers, respectively. Moreover, the thermal stability of the synthesized nanoparticles became almost unwavering above 500 °C. The uncoated iron oxide nanoparticles (IONPs) exhibited the highest thermal stability among all types of synthesized magnetic nanoparticles in this work. 10% weight loss was observed at 183, 141 and 76 °C for the uncoated IONPs, APTES@IONPs and PEG@IONPs, respectively. The Char yields (%) at 900 °C were equal to 61.03, 53.61 and 38.35 % for the uncoated IONPs, APTES@IONPs and PEG@IONPs, respectively. This is because of the existence of the inorganic element that is expected to affect the heating efficiency behavior of the nanoparticles.

**Figure 4:**
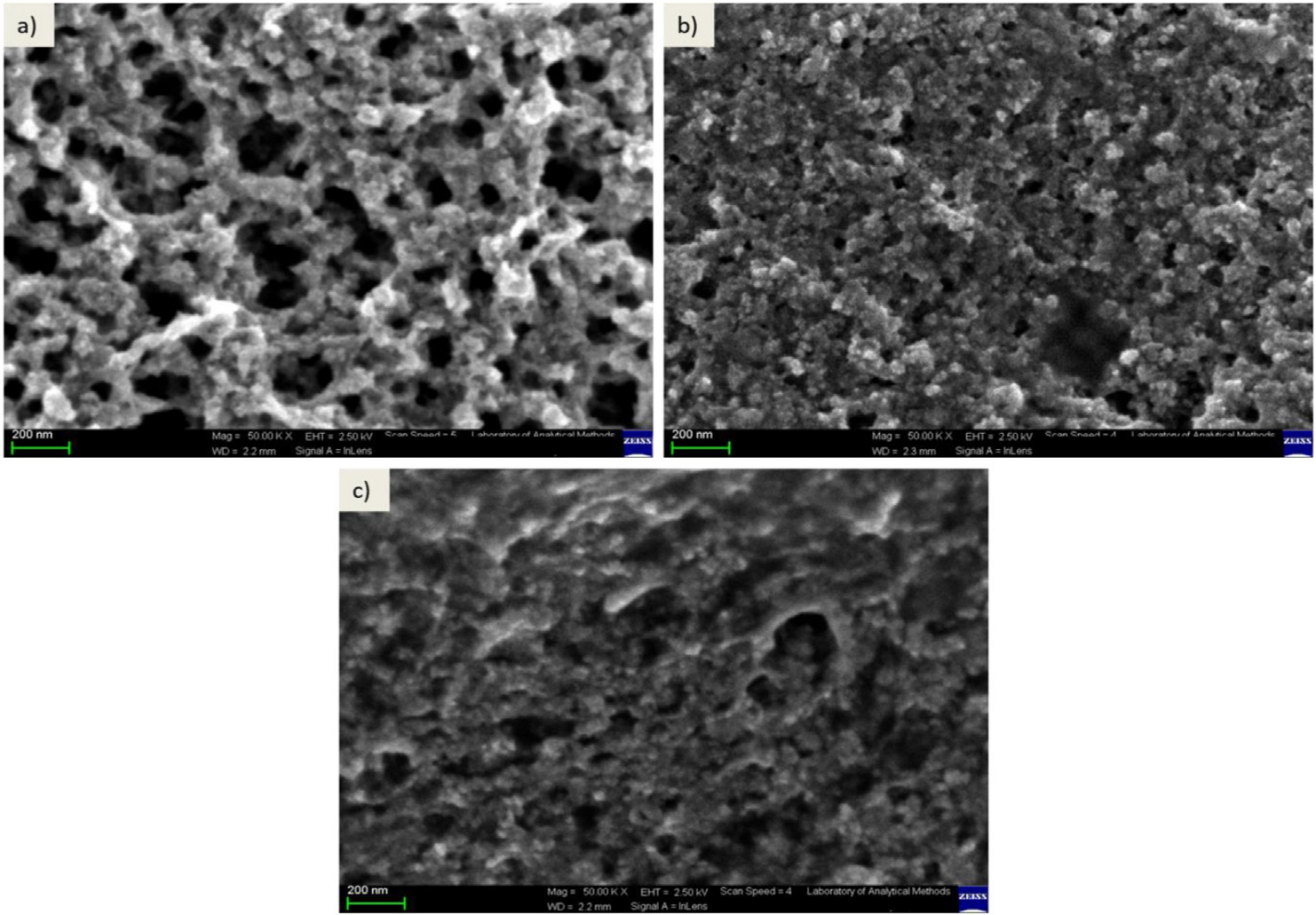
SEM images of a) IONPs, b) APTES@IONPs and c) PEG@IONPs. Scale bars = 200 nm.

The morphology of the uncoated IONPs, APTES@IONPs and PEG@IONPs was investigated by using SEM. According to the SEM images (Figure 4), there are slight differences in the morphology among the synthesized magnetic nanoparticles and broader size distribution. The sizes of the bare IONPs, APTES@IONPs and PEG@IONPs were 26 ± 4, 50 ± 10 and 33 ± 8 nm, respectively. The images indicated the agglomeration of magnetic nanoparticles, attributed to the high surface energy. The size of nanoparticles increased with introducing a shell layer, as this layer served as a protection for better stabilization and particle size control for the nanoparticles [15].

**Figure 5:**
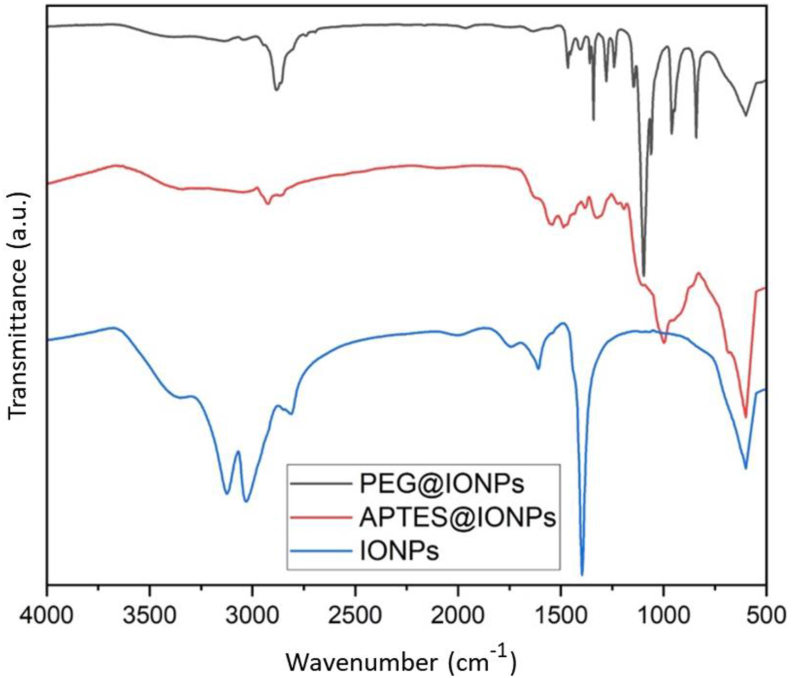
FT-IR results for IONPs, APTES@IONPs and PEG@IONPs.

The FT-IR was used to detect the existing functional groups as shown in Figure 5. The bands at 1090, 800 and 3440 cm^−1^ were owing to asymmetric, symmetric vibrations of Si–O–Si and –NH_2_ for APTES, respectively. The absorption bands at 1200 and 3400 cm^−1^ corresponded to the C–O and O–H in PEG. The absorption band observed close to 580 cm^−1^ confirmed that the Fe–O bond is present in the synthesized uncoated IONPs, APTES@IONPs and PEG@IONPs [16].

**Figure 6:**
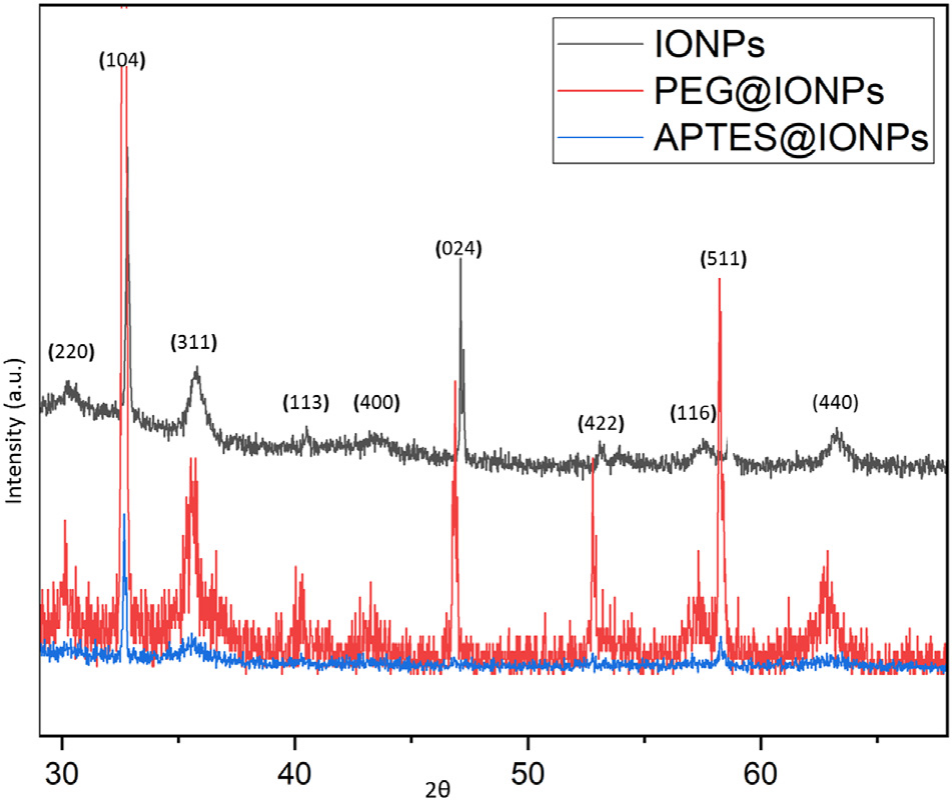
XRD results for IONPs, APTES@IONPs and PEG@IONPs.

The type of magnetic phase and the estimated average grain size were studied using XRD. The peaks along (440), (511), (422), (400), (311) and (220) lattice planes correspond to the standard pattern (reference code: 98-015-8742) for the dominant magnetite nanoparticles phase (Fe_3_O_4_). However, the peaks along (104), (113), (116) and (024) planes correspond to α- Fe_2_O_3_ (Figure 6) [17]. Magnetite phase easily oxidizes at an atmospheric condition in the lack of an encapsulating oxygen block. The peaks for APTES@IONPs and PEG@IONPs were weak and to some extent broad, probable arise from small crystallite and disorder behaviors. The observed grain sizes for IONPs, APTES@IONPs and PEG@IONPs were 23, 40 and 31 nm, respectively and estimated from the Debye–Scherrer equation (Eq. (2)).

(2) $$Dp=\frac{K\lambda }{\beta \text{cos}\theta }$$

where Dp is grain size, *β* is FWHM, *λ* is 1.5406 Å, K is Scherrer constant, and θ is Bragg angle.

**Figure 7:**
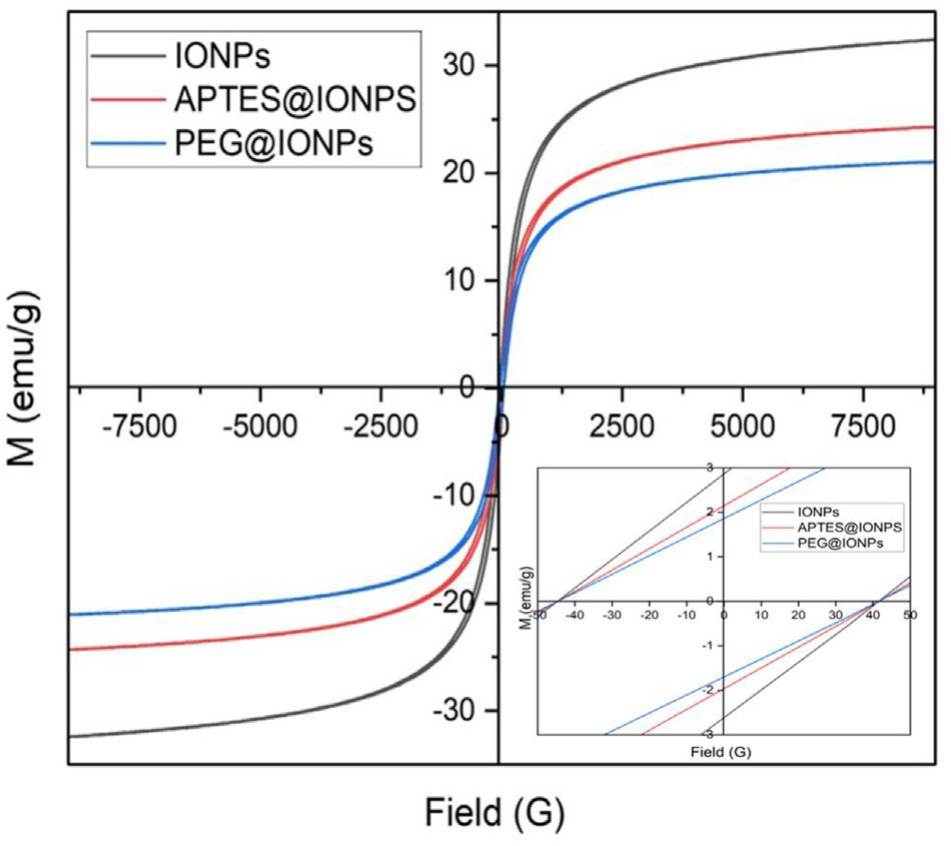
VSM results for IONPs, APTES@IONPs and PEG@IONPs.

The magnetic property of the synthesized IONPs was measured by utilizing VSM. Magnetization saturation (*Ms*) is defined as the maximum magnetization, a point beyond which any further increase in the magnetic field exerts no enhancing influence on the magnetization and saturation is reached. IONPs, APTES@IONPs and PEG@IONPs had *Ms* values of 32.38, 24.28 and 21.04 emu/g, respectively as shown in Figure 7. The lower magnetization saturation of the coated magnetic iron oxide nanoparticles APTES@IONPs and PEG@IONPs arise from the disordered surface spins and the lower crystallinity [18]. The nanoparticles showed low coercivity arising from the soft magnetic nature. It was reported that the size ≈22 nm showed the optimum SAR, for superparamagnetic IONPs [19]. In our study, the prepared nanoparticles gave their rather large diameter without superparamagnetic behavior. Magnetic domains affect by nanoparticles size. The larger size comprise multiple-domain, lead to the minimization of the magnetostatic energy. The magnetic induction heating of IONPs is mainly based on the conditions of externally applied magnetic fields and the properties of nanoparticles.

**Figure 8:**
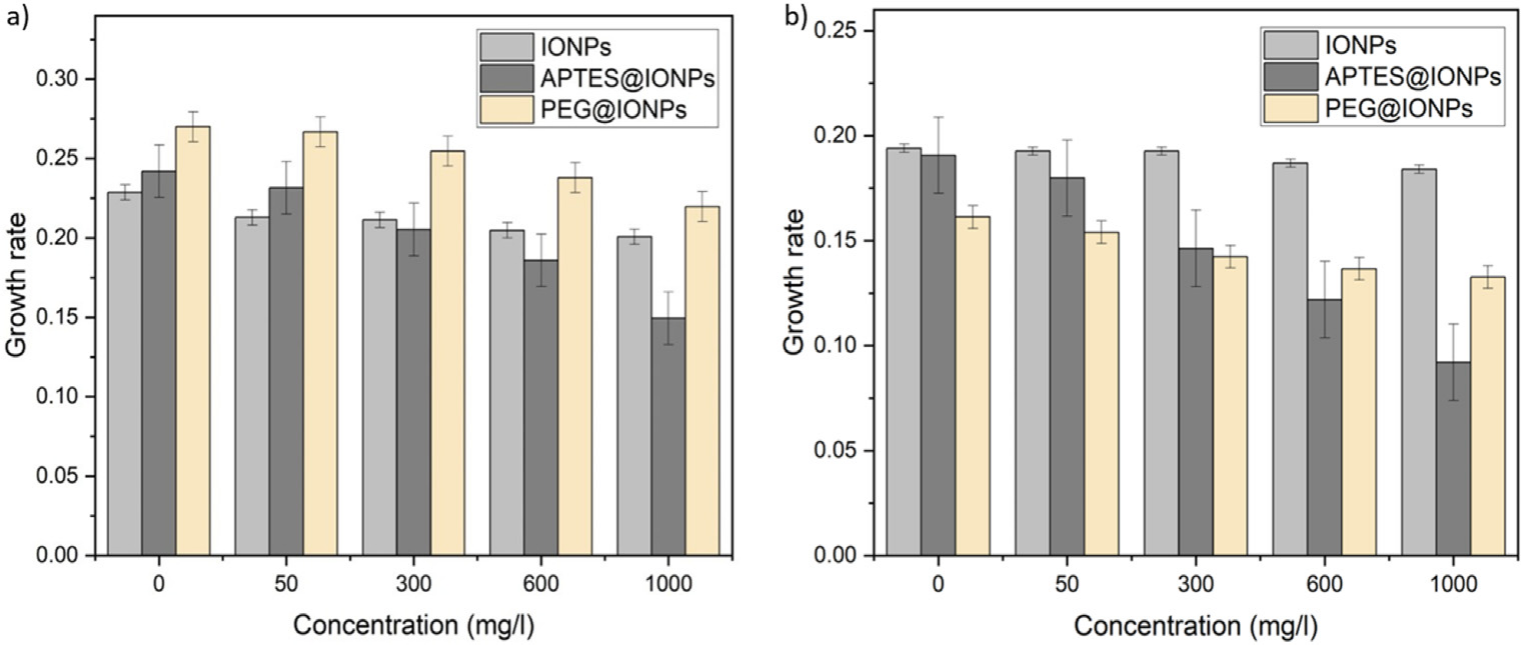
The growth rates (doublings/h) of a) *Escherichia coli* and b) *Staphylococcus aureus*, as observed after 6 h of incubation with IONPs, APTES@IONPs and PEG@IONPs.

Furthermore, the effects of the synthesized IONPs on the bacterial growth of *S. aureus* and *E. coli* were investigated. The unmodified IONPs exhibited no remarkable impact on the growth rates of the two types of bacteria. However, for APTES@IONPs and PEG@IONPs, the growth rates of both the bacterial species were observed to be decreased with the increase in nanoparticles concentration (Figure 8). The growth rate of *E. coli* dropped to 0.15 doublings/h with APTES@IONPs (1 g/L), while the growth rate was 0.22 doublings/h when with PEG@IONPs (1 g/L). At all concentrations, APTES@IONPs exhibited a greater influence on the growth rate of *E. coli* when compared to the effect of PEG@IONPs. Upon exposure to APTES@IONPs and PEG@IONPs, the growth rates of *S. aureus* were 0.09 and 0.13 doublings/h, respectively. These results indicated that PEG@IONPs could be considered safe and non-toxic for use in biomedical applications. APTES@IONPs, on the other hand, exhibited a degree of antimicrobial activity, rendering these suitable for other bio-applications where bacterial growth is undesirable, such as using as hyperthermic agents. Several reports have evaluated the toxic impacts of magnetite nanoparticles on eukaryotic organisms and reported the negligible toxicity of surfactant-modified magnetite nanoparticles [20, 21]. The biocompatibility and no-toxicity of IONPs make them suitable for application in the biomedical field. It is, however, noteworthy that the coating material used in the preparation of nanoparticles strongly affected biocompatibility [22, 23]. The coating of the IONPs surface protects the magnetic core from the oxidative environments, thereby enhancing the compatibility and stability and also improving the efficiency of the tumor-targeting.

**Figure 9:**
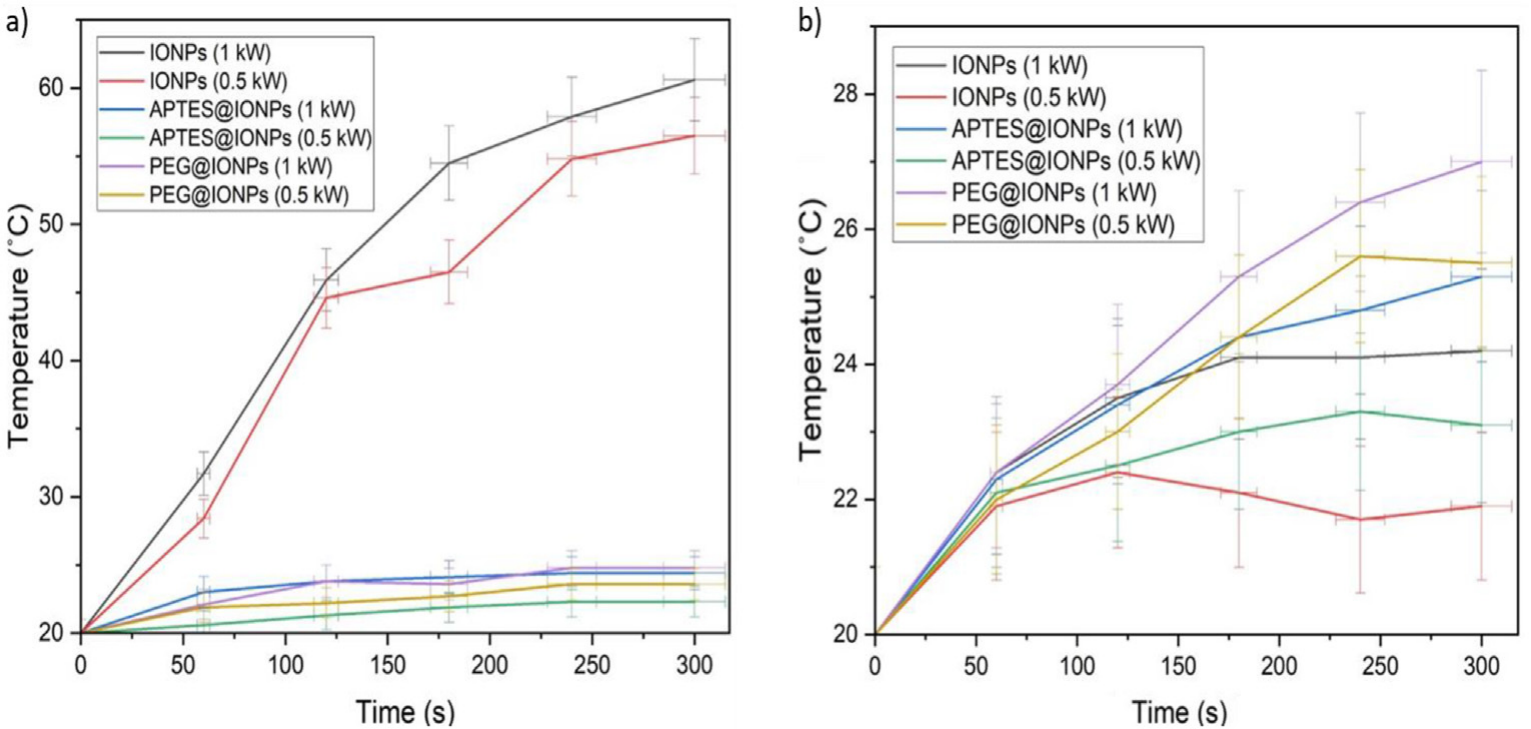
Heating efficiencies of IONPs, APTES@IONPs and PEG@IONPs under the magnetic field power values of 1 kW and 0.5 kW and a frequency of 142 kHz, in (a) water and (b) ethanol as carriers.

IONPs generated localized heat energy under the alternating magnetic field. This increased energy forms the basis of the application of magnetic induction heating properties in cancer therapy [24, 25, 26, 27]. Next, the influences of viscous carriers (ethanol and water) and the magnetic field power (1 and 0.5 kW) with a frequency of 142 kHz on the heating profile of the synthesized IONPs were investigated. Regarding the effective application of hyperthermia, nanoparticles must fulfill the criteria of lower dose with a high *Ms* value. As depicted in Figure 9, a quasi-adiabatic reign, in which no heat exchange takes place between specimen and medium. The temperature elevated linearly with time and then the increase in temperature decelerated gradually until reaching the saturation stage. Thereafter, the temperature did not increase any further due to the occurrence of thermal equilibrium which is defined as a point at which heating rate equals cooling rate [24]. The specimens became heated faster, and the heating rate rose with the power increase (Figure 9). After 300 s of AMF application at a higher magnetic field power of 1 kW, the heating rate increased for IONPs, APTES@IONPs and PEG@IONPs, and the corresponding temperature elevations were 40.6, 4.4, and 4.8 °C, respectively. After 300 s of AMF application with a lower magnetic field power of 0.5 kW, the heating rate increased for IONPs, APTES@IONPs and PEG@IONPs, and the temperature elevations were 36.5, 2.3 and 3.6 °C, respectively. As a consequence, the heating efficiency increased by the increase in the magnetic field power under constant frequency. Furthermore, for an effective hyperthermia-based treatment, the temperature of the cancer cells must be elevated to the range of 43–45 °C. Therefore, a temperature elevation of 6–8 °C is required. Iron oxide nanoparticles have the characteristic of delivering high heating performance at a low dose, thereby enabling rapid hyperthermia-based treatment. When comparing the heating efficiencies of IONPs, APTES@IONPs and PEG@IONPs under the same magnetic field and frequency, it was observed that IONPs exhibited the highest efficiency among all three. The enhanced high magnetization saturation of IONPs significantly improved their heating performance. The use of ethanol as a solvent resulted in an almost constant heating rate with the time of exposure, showing that ethanol hindered the transfer of convective heat under the alternating magnetic field. The heat dissipation resulted fundamentally from the magnetic moment's relaxation process, either Néel relaxation or Brownian relaxation.

**Figure 10:**
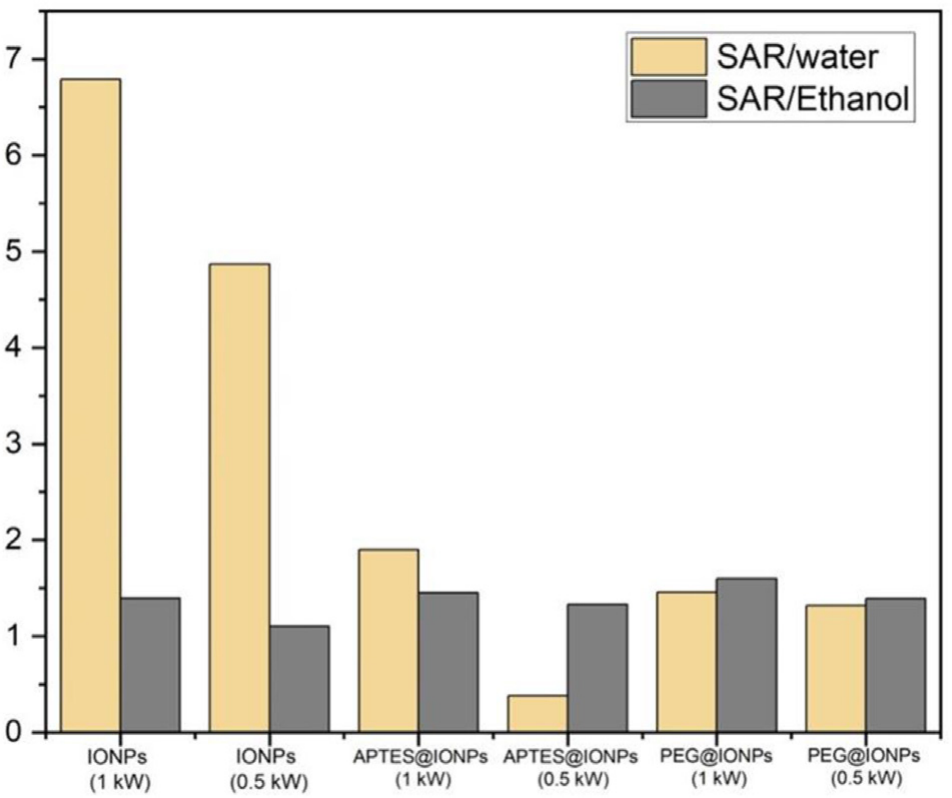
SAR values of IONPs, APTES@IONPs and PEG@IONPs.

To be safe for human exposure, the product of the strength and the frequency of the AMF must be maintained below a threshold safety value of 5 × 10^9^ A/m.s. Moreover, SAR is a function of the magnetic field strength. Therefore, the values of SAR increase by the increase in the applied power. The heating efficiency of the particles synthesized in the present study could be enhanced by using a power of 1 kW and a frequency of 142 kHz. A comparative analysis of the SAR values of IONPs, APTES@IONPs and PEG@IONPs is presented in Figure 10. Under the same magnetic field and frequency, IONPs exhibited higher SAR values compared to APTES@IONPs and PEG@IONPs, indicating a better hyperthermic response of IONPs. The highest value of SAR of 6.79 W/g was obtained for IONPs at 142 kHz as frequency and 1 kW as power. The lowest SAR value of 1.1 W/g was obtained for MNPs at 0.5 kW as power and 142 kHz as frequency. There was a distinct decline in SAR from 6.79 W/g (water as carrier) to 1.3 W/g (ethanol as carrier). As evident in Eq. (3) for SAR, SAR was also influenced by Ms. The modulation of shape and size of IONPs could result in altered magnetic behavior, induced Ms, and further enhance the heating efficiency of IONPs.

(3) $$SAR=\frac{\pi \cdot \mu_{0}\cdot \chi ”\left(f\right)\cdot H^{2}\cdot f}{\rho_{MNPs}\cdot \varphi },\;\chi ”\left(f\right)=\frac{\mu_{0}M_{s}^{2}V}{3k_{B}T}\frac{\omega \tau_{R}}{\left(1+\omega^{2}\tau_{R}^{2}\right)}$$

*f*: the frequency, *H*: the field strength, *ρ*_*MNPs*_: the density of nanoparticles, *φ*: the volume fraction, *μ*_*0*_: the permeability and *χ”*: the susceptibility. *M*_*s*_: the saturation magnetization, *V*: the volume of the MNPs, *τ*_*R*_: the time of relaxation, *k*_*B*_: the Boltzmann's constant and *ω* = *2πf* is the sweep rate of AMF.

Table 1, comparison between materials reported in the literature and the current research [28, 29, 30, 31, 32, 33]. The heating efficiency is influenced by several factors, such as magnetic behaviors, the conditions of a magnetic field, the size of nanoparticles and the coating layer. Therefore, using an appropriate way for reaching the target temperature when synthesizing the magnetic nanoparticles is necessary [34, 35]. The maximum expected SAR values matched the size between 70 and 100 nm [36]. The range of single-domain size range is 20–70 nm to improve the loss of power value [37]. The reported SAR values in the previous studies are in the range of 10–100 W/g for frequency 400 kHz and magnetic field ​10 kA/m [38]. IONPs - citrate exhibited a high Ms value (57 emu/g) and a high SAR value within a short duration as the magnetic field increased [39]. In another study, a high value of SAR (150 W/g) was reached at *f* = 205 kHz and *H* ​= ​20 mT [40]. Furthermore, IONPs - chitosan exhibited higher SAR (119 W/g) compared to the bare IONPs [41]. In a different work, the SAR value was altered from being almost zero for IONP (4.1 nm) to 76 W/g for the IONP- rhamnose (35 nm) [42]. The two kinds of relaxations also rely on the size of the magnetic particles. In general, when IONPs are less than 20 nm in size, Néel relaxation dominates [43]. The magnetic fluid MFL AS is a formulation that comprises IONPs - amino silane with a size of 15 nm, which is approved for a clinical trial. The desired field for reaching the base temperature at f: 100 kHz and H: varying from 0 to 18 kA/m [44].

**Table 1:** Comparison between particles reported in the literature and this work.

Magnetic material	Surface layer	Size (nm)	M_s_ (emu/g)	SAR (W/g)	Ref.
Fe_3_O_4_	PEG	19	80	2452	[28]
Fe_3_O_4_	Silica	10	3	20	[29]
Fe_2_O_4_	Silica	14.8	2.5	20	[30]
Fe_3_O_4_	-	7.5	10	15.5	[31]
CoFe_2_O_4_	PEG	7	32.3	11	[32]
Mn_1+x_ Fe_2-2X_Ti_1+x_ O_4_	-	20	8	2	[33]
Fe_3_O_4_	-	26	32.38	6.79	Current study
Fe_3_O_4_	PEG	33	21.04	1.4	Current study
Fe_3_O_4_	APTES	50	24.28	1.9	Current study

## 4 Conclusions

Functionalized IONPs, APTES@IONPs and PEG@IONPs were synthesized via an ultrasonic-assisted co-precipitation technique. The existence of APTES or PEG layer on the coated IONPs surface led to disordered surface spins, allowing lower Ms compared to that observed for bare iron oxide nanoparticles. APTES@IONPs exhibited a degree of antimicrobial activity, rendering it suitable for utilization in the bio-applications in which bacterial growth is undesirable, such as the use as hyperthermic agents. IONPs attained the highest value of SAR (6.79 W/g) when using water as the dispersing medium. The hydrodynamic volume and the viscosity of particles hinder the Brownian relaxation, causing the inhibition of IONPs rotation in the medium. The synthesized nanoparticles in the present study could be applied as agents of hyperthermia-based processes. Nonetheless, further investigation regarding hyperthermia-based drug release is warranted.

## Declarations

### Author contribution statement

Mohamed S. A. Darwish: Conceived and designed the experiments; Performed the experiments; Analyzed and interpreted the data; Wrote the paper.

L.M. Al-Harbi: Conceived and designed the experiments; Analyzed and interpreted the data; Contributed reagents, materials, analysis tools or data; Wrote the paper.

### Funding statement

This research did not receive any specific grant from funding agencies in the public, commercial, or not-for-profit sectors.

### Data availability statement

Data included in article/supplementary material/referenced in article.

### Declaration of interests statement

The authors declare no conflict of interest.

### Additional information

No additional information is available for this paper.
